# Power-Dependent Investigation of Photo-Response from GeSn-Based p-i-n Photodetector Operating at High Power Density

**DOI:** 10.3390/ma15030989

**Published:** 2022-01-27

**Authors:** Chiao Chang, Hung-Hsiang Cheng, Gary A. Sevison, Joshua R. Hendrickson, Zairui Li, Imad Agha, Jay Mathews, Richard A. Soref, Greg Sun

**Affiliations:** 1 Center for Condense Matter Sciences and Graduate Institute of Electronics Engineering, National Taiwan University, Taipei 106, Taiwan; alicechang.us@gmail.com; 2 Sensors Directorate, Air Force Research Laboratory, Wright-Patterson AFB, OH 45433, USA; sevisong1@udayton.edu (G.A.S.); joshua.hendrickson.4@us.af.mil (J.R.H.); 3 Department of Electro-Optics and Photonics, University of Dayton, Dayton, OH 45469, USA; liz001@udayton.edu (Z.L.); iagha1@udayton.edu (I.A.); 4 Department of Physics, University of Dayton, Dayton, OH 45469, USA; jay.mathews@udayton.edu; 5 Department of Engineering, University of Massachusetts Boston, Boston, MA 02125, USA; soref@rcn.com (R.A.S.); Greg.Sun@umb.edu (G.S.)

**Keywords:** GeSn, photodetector, photo-response

## Abstract

We report an investigation on the photo-response from a GeSn-based photodetector using a tunable laser with a range of incident light power. An exponential increase in photocurrent and an exponential decay of responsivity with increase in incident optical power intensity were observed at higher optical power range. Time-resolved measurement provided evidence that indicated monomolecular and bimolecular recombination mechanisms for the photo-generated carriers for different incident optical power intensities. This investigation establishes the appropriate range of optical power intensity for GeSn-based photodetector operation.

## 1. Introduction

With the expansion of group-IV elements to include Sn, the prospect of achieving efficient group-IV photonic devices, such as photodiodes (PDs) and light emitting diodes (LEDs) that can be integrated on Si substrates in a CMOS compatible process, becomes much brighter. While, recently, III-V heterogeneous integration has seen commercial success, lasers and amplifiers are generally more critical than detectors, especially since III-V processing in a CMOS foundry is undesirable [1]. In fact, recent breakthroughs in GeSn-based p-i-n PDs and LEDs have demonstrated excellent optical properties for important applications in the near- and mid-infrared regions [2,3,4,5,6,7,8]. With the continuous performance improvement of both PDs [9,10,11,12,13,14,15,16,17,18] and LEDs [19,20,21,22,23], the responsivities of GeSn-based PDs have been shown to be comparable with those of commercial extended wavelength InGaAs PDs [24], and their absorption wavelength range has been extended to well beyond 2 μm, which is desirable for applications in optical communications and night vision [9,25]. The properties of GeSn PDs, such as photo-responsivity, have been studied by many research groups with respect to temperature dependence [14,24], Sn-composition dependence [11,24], as well as structural dependence [26]. One aspect of the GeSn PDs that has not been studied is their performance under various incident power conditions. There is, however, a need for high-power, high-gain and high-speed p-i-n PDs functioning as efficient RF photonic links. InGaAs PDs have been used to fulfill this need to some extent, but InGaAs is known to be a poor thermal conductor—a severe material limitation. With almost 10 times better thermal conductivity, PDs made of GeSn are better suited for high-frequency RF applications. In this investigation, we perform a study of the power-dependence of photo-responsivity in GeSn PDs over their active absorption wavelength range. The experimental results, combined with the modeling of external quantum efficiency, clearly show the effect of different carrier recombination mechanisms at work under different incident power intensities.

## 2. Materials and Methods

**Figure 1:**
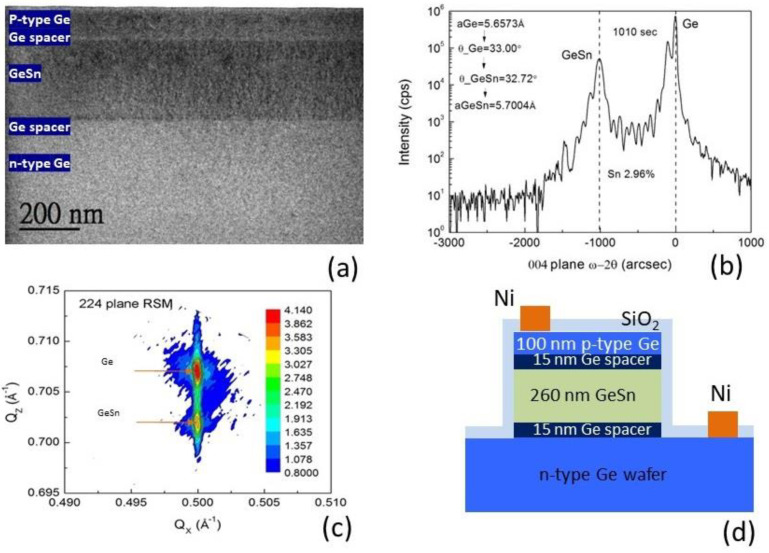
(**a**) XTEM image of the MBE-grown GeSn sample on n-type Ge wafer. (**b**) XRD rocking curve scan on the (004) plane. The Sn composition of ~3% is measured. (**c**) (224) reciprocal space mapping of the GeSn sample. (**d**) Cross sectional view of the schematic structure of the fabricated GeSn-based PD.

The sample was grown using solid source molecular beam epitaxy (MBE) with a low temperature growth technique. The diode structure was grown on an n-type Ge (001) wafer with a resistivity of 1 Ω·cm, consisting of the following layers: (i) undoped layers of Ge-spacer/Ge_1−x_Sn_x_/Ge-spacer with thickness of 15/260/15 nm and (ii) a p-type doped Ge layer of 100 nm. The thicknesses of these layers were measured with cross-sectional transmission electron microscopy (XTEM), as shown in Figure 1a. The Sn content in the GeSn layer was determined by high-resolution X-ray diffraction measurement and was found to be 3%, as shown in Figure 1b. As well as the (004) X-ray measurement, (224) reciprocal space mapping was also performed, as shown in Figure 1c. The measurement was performed with a large probing spot size of 0.5 mm × 10 mm to estimate the strain status in the epilayer. The plot shows that the diffraction peaks of the GeSn and Ge layers were located at the same $Q_{x}$, which indicates that the GeSn layer complied with the lattice structure of the Ge wafer. That is, the GeSn layer was nearly fully strained and was consistent with X-ray measurement.

The sample was processed into a circular mesa using the dry etching technique with a reactive ion etcher (RIE). A p-i-n diode was fabricated by etching down to the n-type Ge wafer. Diodes with diameters of 300 μm were fabricated. After processing, a thin SiO_2_ layer with a thickness of 200 nm was deposited using plasma-enhanced chemical vapor deposition. During the SiO_2_ layer deposition, the wafer temperature was set at 300 °C, which is below the critical temperature for the epilayer to relax and the Sn to segregate. This layer served as an insulating layer to prevent current flow from the P-type contact to the n-type contact through the sidewall surface of the diode under the applied voltage. After the processing, Ni was deposited as the electrical contact. The schematic plot of the diode is shown in Figure 1d.

One advantage in the fabrication approach chosen here is that the Ge and GeSn layers are grown in situ using MBE, resulting in a better interface between the layers. This growth method helps avoid deleterious defects that can affect transport or absorption, as seen in other growth methods where the Ge and the GeSn are grown in separate chambers. An additional advantage of the proposed approach is that sandwiching of GeSn between layers of Ge leads to some optical confinement in the GeSn layer, as well as enhancement of absorption at certain wavelengths due to interference. By varying layer thicknesses and composition, one can enhance the performance of the detector over a narrow band without having to deposit more dielectric layers. This simplifies the fabrication process.

**Figure 2:**
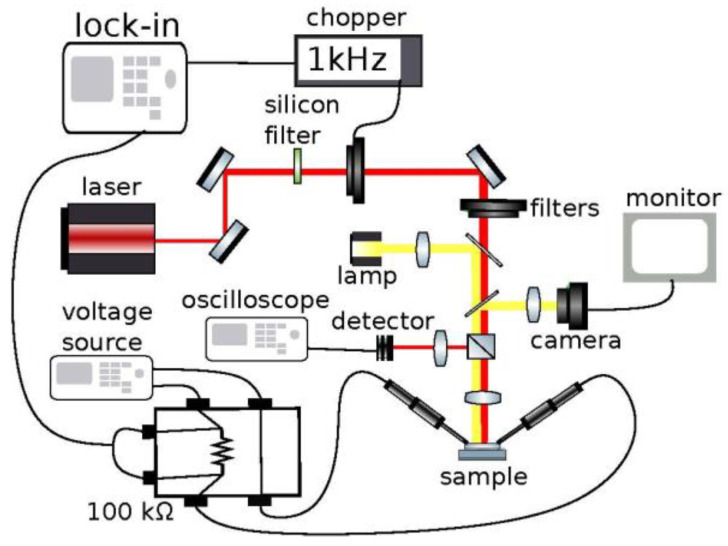
Schematic of the optical measurement setup.

Photo-response measurements with front-side illumination were performed with a <10 ns tunable laser at 1 kHz repetition rate. Neutral density filters were used to adjust the power incident on the device and a beam splitter was used to send half the power to a detector and half the power to the device. The beam was then focused down into a spot on the detecting portion of the device. Measurements were taken over a wavelength range of 1500 nm to 1800 nm. The average power incident on the device was measured to range from approximately 40 μW to 3.5 mW at the laser’s peak wavelengths. Five separate power level measurements were taken at each wavelength. The device was held at a bias voltage of 5 V and was placed in series with a 1 kΩ resistor. Voltage measurements were taken across the resistor using a lock-in detection technique. For time-resolved measurements the lock-in voltage response was monitored with a GHz oscilloscope using laser powers in the range of 5 μW to 100 μW. A schematic of the optical measurement setup is displayed in Figure 2.

## 3. Results

**Figure 3:**
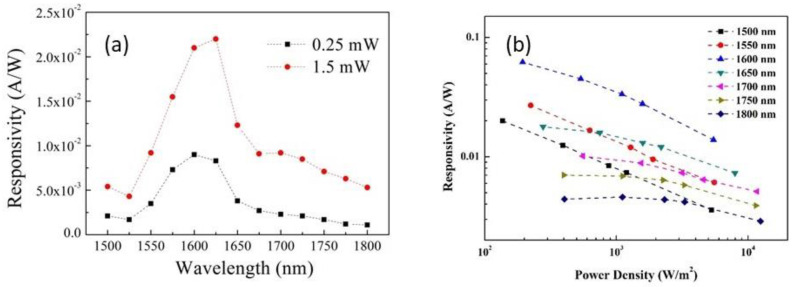
(**a**) Responsivity vs. incident wavelength measured at incident laser powers of 0.25 and 1.50 mW. (**b**) Responsivity vs. incident power density at different wavelength from 1500 nm to 1800 nm.

The responsivity of the GeSn PD under different incident laser power intensities in the wavelength range from 1500 nm to 1800 nm was measured and the results are shown in Figure 3. The measured responsivity vs. the incident wavelength, ranging from 1500 to 1800 nm at two incident laser powers of 0.25 and 1.50 mW, exhibited a peak around 1625 nm, as shown in Figure 3a. This behavior can be understood in terms of the optical absorption which is stronger at shorter wavelengths because the optical transition occurs between deeper conduction and valence-band states with higher densities of states, but at the same time, the number of photons that can be absorbed is less at shorter wavelengths for fixed incident power. The measured responsivity vs. the incident power density is plotted in Figure 3b. The measured responsivity was comparable with the published results of GeSn PDs of similar Sn composition of 2.8% [5] and 3.0% [6], but smaller than the theoretical prediction of achievable performance [7]. There were, however, several different behaviors at different wavelengths: (a) at the wavelengths of 1500 and 1550 nm, responsivity reduced exponentially as the power intensity increased, (b) at wavelengths from 1600 to 1700 nm, the responsivity decayed slowly in the lower power intensity range, and then the rate of decay picked up in the higher power intensity range, and (c) at wavelengths over 1750 nm, the responsivity remained nearly constant in the lower power density range, and then reduced gradually as the power density increased. While the general trend of reduction in responsivity with the increase in incident power can be understood as the result of increase in carrier recombination rate for higher carrier density created by the higher incident power, the different rates of responsivity reduction in different power intensity ranges, however, have not been observed before in GeSn PDs and the mechanism that leads to this behavior is not well understood. We conduct an analysis below to reveal the mechanism.

## 4. Discussion

To interpret the observations, there are two kinds of mechanisms to be considered: extrinsic (monomolecular) and intrinsic (bimolecular) recombination. Extrinsic recombination requires only one type of carrier to be captured by one type of impurity center. This type of recombination can be described by the SRH model where the average lifetimes for electrons and holes are defined as $\tau_{n}=1/B_{n}N_{t}$ and $\tau_{p}=1/B_{p}N_{t},\;\;$ in which $B_{n}$ and $B_{p}$ are the trapping coefficients, respectively, and $N_{t}$ is the density of trap states. The monomolecular recombination effect is therefore limited by the impurity density in the material. When the carrier concentration is much higher than the impurity concentration, the effect of intrinsic recombination, that involves both a free electron and a free hole (including radiative recombination), must be taken into account and this effect is largely responsible for the reduction of responsivity and, therefore, photocurrent. Generally, in Ge-based material systems, extrinsic recombination processes dominate over intrinsic recombination [27]. The intrinsic bimolecular recombination is generally characterized by the minority lifetimes for electrons and holes as $\tau_{n}=1/B_{r}p_{0}$ and $\tau_{n}=1/B_{r}n_{0}$ where $n_{0}$ and $p_{0}$ are the electron and hole concentrations, respectively, and $B_{r}$ is the direct recombination coefficient.

**Figure 4:**
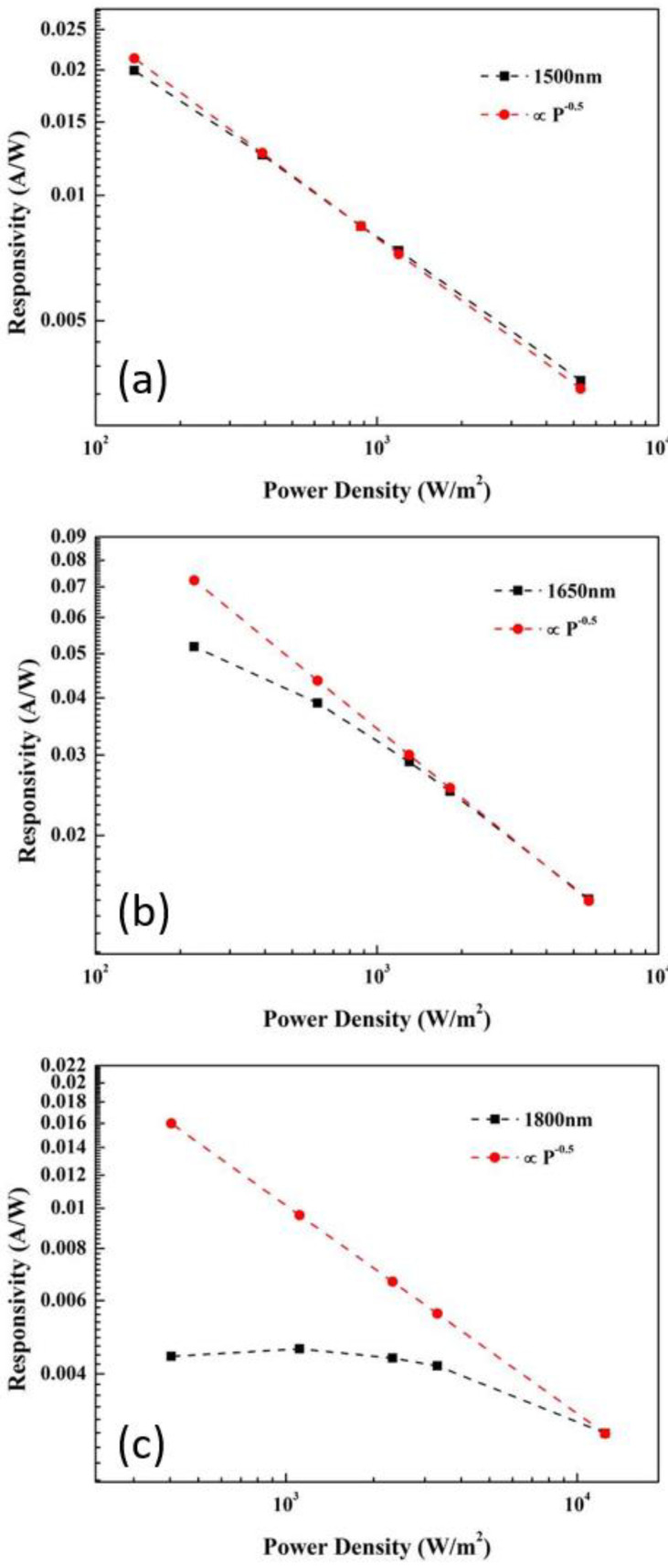
Power-dependent responsivities with incident laser wavelength of (**a**) 1500 nm, (**b**) 1650 nm, and (**c**) 1800 nm.

The conventional treatment of optical absorption has always assumed that the photocurrent is proportional to the incident optical power so that the responsivity is not changed with different incident optical power. However, this is clearly not the case here. In our study under high incident laser power, at 1500 nm, as shown in Figure 4a, the responsivity decreased with the incident power intensity P as P^−0.5^. This behavior demonstrates the dominance of bimolecular recombination which happens at higher power intensities because of the higher photo-generated carrier density. Since the absorption coefficient α for GeSn is higher for shorter wavelengths, carrier density generated with shorter wavelength incident light could be higher at lower optical power intensity than that generated with light of longer wavelength at higher power intensity. Figure 4a suggests that, for 1500-nm incident light with α ≈ 7000 cm^−1^, the bimolecular recombination effect dominates over the whole range of power density from 100 to 5000 W/m^2^. With 1650-nm incident light with α ≈ 4000 cm^−1^, significantly lower than 7000 cm^−1^, an exponential decay following P^−0.5^ dependence was still observed at a power intensity higher than 1 × 10^3^ W/m^2^, as shown in Figure 4b. Below 1 × 10^3^ W/m^2^, however, the responsivity decreased more slowly with incident optical power. Figure 4c shows the power-dependent responsivity of 1800-nm light with α ≈ 150 cm^−1^, exhibiting a clear departure from the P^−0.5^ dependence.

The highest responsivity exhibited by the device occurred at a wavelength of 1625 nm. The active GeSn layer has a higher index of refraction than the Ge layers, which leads to reflections at the boundaries, thereby causing interference effects. We modeled the index of refraction of GeSn using the method presented by Tran et al. [28] and used the transfer matrix method to calculate the absorption in the GeSn layer. The calculated absorption spectrum showed a peak at 1625 nm; thus, the device response was highest at that wavelength due to the increased optical absorption.

**Figure 5:**
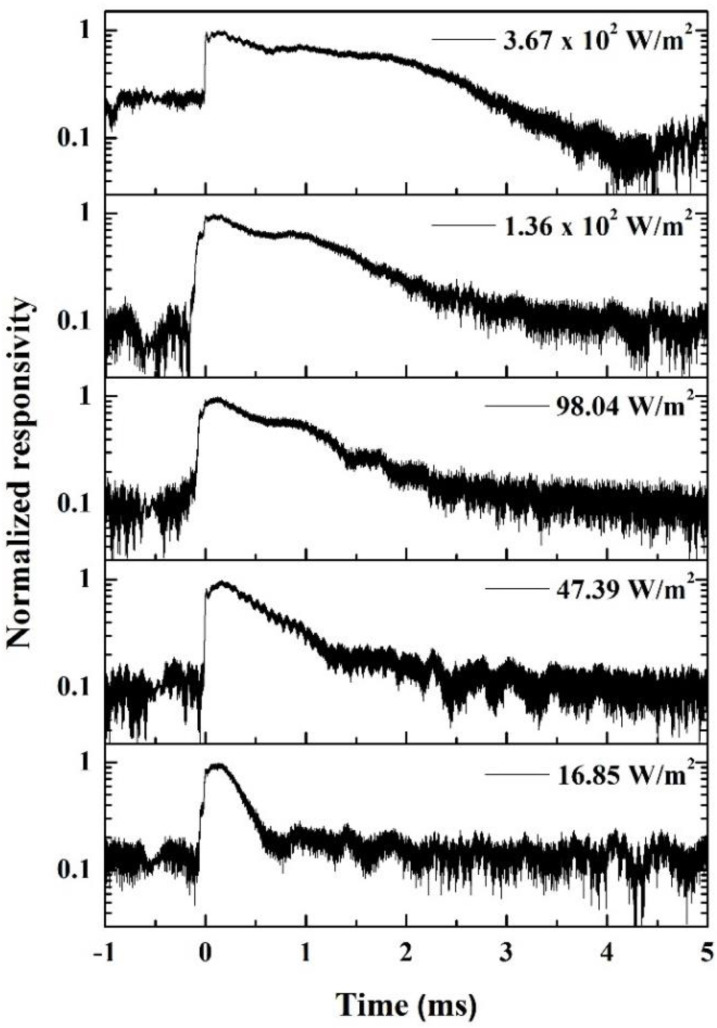
Time-resolved 1550 nm responsivity measurement with different laser power densities.

To further investigate the transition from monomolecular recombination to bimolecular recombination, we conducted power-dependent time-resolved responsivity measurement with a 1550 nm incident laser pulse of 10 ns. The temporal responsivity is shown on a log-scale in Figure 5. At low incident power intensities of 16.85 and 47.39 W/m^2^, the responsivity exhibited exponential decay, which is a characteristic of monomolecular recombination. At higher incident power intensities beyond 98.04 W/m^2^, the initial hyperbolic decay is due to bimolecular recombination, and the following exponential decay is from monomolecular recombination. This result is consistent with Figure 4a that the bimolecular recombination effect comes with lower power density than 98.04 W/m^2^, the first data point in Figure 4a. In order to obtain optimal photo-responsivity performance in GeSn PDs, the appropriate operation incident power intensity should therefore not exceed ~100 W/m^2^ at 1.55 μm wavelength. 

The detected pulses have a time scale in the milliseconds, which would limit operation to the kHz frequency range. However, this device has not been optimized to achieve high frequency operation. The limitation in speed is due to the recombination processes, some of which can be controlled. SRH recombination is related to the number of traps due to defects in the material, which could be introduced during the growth of the active GeSn layer. Increasing the defect density should lead to an increase in the SRH recombination rate, thereby reducing the effective recombination lifetime in the active layer. This could also reduce the effect of bimolecular recombination, possibly leading to improved performance at higher power.

## 5. Conclusions

In summary, the power-dependence of the GeSn-based PD was studied for the first time. We used a tunable laser with a range of incident light power to study the power dependence of the responsivity of the GeSn-based PD illuminated at different wavelengths. An exponential increase in photocurrent, and an exponential decay of responsivity with increase in optical power density, were observed in the higher incident power density range. The power-dependent behavior in a GeSn-based PD can be explained by different recombination mechanisms that dominate within different power density ranges. Time-resolved measurements revealed monomolecular and bimolecular recombination of excited carriers at play in the lower and higher incident power density ranges, respectively. This study establishes the incident power density range for the GeSn PD to achieve its optimal responsivity.

## Data Availability

Data presented in this study is available on request from the corresponding author.
